# The value of chest CT for prediction of breast tumor size: comparison with pathology measurement

**DOI:** 10.1186/1477-7819-11-130

**Published:** 2013-06-06

**Authors:** Su Joa Ahn, Young Saing Kim, Eun Young Kim, Heung Kyu Park, Eun Kyung Cho, Yoon Kyung Kim, Yon Mi Sung, Hye-Young Choi

**Affiliations:** 1 Department of Radiology, Gachon University GilHospital, 1198, Guwol-dong, Namdong-gu, Incheon, 405-760, Republic of Korea; 2 Division of Hematology/Oncology, Department of Internal Medicine, Gachon University Gil Hospital, Incheon, Republic of Korea; 3 Department of Surgery, Gachon University Gil Hospital, 1198, Guwol-dong, Namdong-gu, Incheon, 405-760, Republic of Korea

**Keywords:** Breast cancer, Chest CT, Tumor size, Pathology

## Abstract

**Background:**

Little information is available on the use of chest computed tomography (CT) to predict breast tumor size in breast cancer, despite the fact that chest CT examinations are being increasingly used. The purpose of this study was to evaluate the value of chest CT for predicting breast tumor size using pathology measurements as reference standards.

**Methods:**

Tumor sizes (defined as greatest diameter) were retrospectively measured on the preoperative chest CT images of 285 patients with surgically proven unifocal, invasive breast carcinoma. Greatest tumor diameters as determined by chest CT and pathologic examinations were compared by linear regression and Spearman’s rho correlation analysis. Concordance between CT and pathology results was defined as a diameter difference of <5 mm. Subgroup analyses were also performed with respect to tumor size (<20 mm or ≥20 mm) and histological subtype (invasive ductal carcinoma(IDC) or non-IDC).

**Results:**

CT and pathology measured diameters were found to be linearly related (size at pathology = 1.086 × CT determined tumor size - 1.141; Spearman’s rho correlation coefficient = 0.84, *P*<0.001). Most tumors (*n* = 228, 80.0%) were concordant by chest CT and pathology, but 36 tumors (12.7%) were underestimated by CT (average underestimation, 11 mm; range, 6–36 mm) and 21 tumors (7.4%) were overestimated (average overestimation by CT, 10 mm; range, 6–19 mm). The concordance rate between the two sets of measurements was greater for tumor of <20 mm and for IDC (*P*<0.001 and *P* = 0.011, respectively).

**Conclusions:**

Tumor size by chest CT is well correlated with pathology determined tumor size in breast cancer patients, and the diameters of the majority of tumors by chest CT and pathology differed by <5 mm. In addition, the concordance rate was higher for breast tumors of <20 mm and for tumors of the IDC histologic subtype.

## Background

Breast cancer is the most common malignancy in women, and accounts for 15% of all cancers [1]. The accurate estimation of tumor size and extent in breast cancer is essential for surgical planning and minimizing local recurrence after surgery [2,3], and recently, chest computed tomography (CT) has been used to detect pulmonary and hepatic metastasis in patients with breast cancer before surgery. However, chest CT screening is not currently recommended in operable and asymptomatic patients with breast cancer [4,5], although the use of chest CT examinations in breast cancer patients is undoubtedly increasing. Furthermore, a review of the literature revealed that no study has yet addressed the use of chest CT for determining breast tumor size. Accordingly, the purpose of this study was to assess the value of chest CT for determining breast tumor size using pathology measurements as reference standards.

## Methods

### Patients

We retrospectively searched archived records in a practice specializing in breast surgery from April 2010 to January 2012 for breast cancer patients that underwent chest CT before surgical treatment. The inclusion criteria adopted were newly diagnosed, biopsy-proven, unifocal breast cancer with a positive chest CT scan. Finally, 285 patients (mean age, 48.79 years; range, 20–79 years) were included. Baseline characteristics of the study cohort are provided in Table 1.

**Table 1:** Clinicopathologic characteristics of the study subjects

**Characteristics**	**Tumors <20 mm on CT ( *n *= 139)**	**Tumors ≥20 mm on CT ( *n *= 146 )**	***P *values**
Age (years)^a^	50.1±9 (31–78)	47.5 ± 10 (20–79)	0.249^b^
*Stages*			
IA	106	9	
IB	0	0	
IIA	28	74	
IIB	0	29	
IIIA	1	19	0.724^c^
IIIB	1	1	
IIIC	3	14	
*T stages*			
T1	138	13	0.267^c^
T2	0	124	
T3	0	8	
T4	1	1	
*N stages*			
N0	107	81	
N1	28	33	0.467^c^
N2	1	18	
N3	3	14	
ER status			
Positive	95	81	0.164^c^
Negative	44	65	
PR status			
Positive	70	62	0.020^++^
Negative	69	84	
*HER-2 amplification*			
Positive	75	79	0.113^c^
Negative	64	67	
Unknown			
Histopathologic type			
Invasive ductal carcinoma	119	126	
Invasive lobular carcinoma	6	7	
Others^d^			
Histologic grade^e^			
Grade 1	26	8	0.091^c^
Grade 2	82	73	
Grade 3	31	65	
Unknown			
Operation type			
Mastectomy	17	33	0.051^c^
Breast conserving surgery	122	113	

^a^ Mean values with standard deviations (numbers in parentheses are ranges).

^b^ Two-sample *t*- test.

^c^ Chi-squared test.

^d^ Others including invasive papillary carcinoma (*n*= 6), invasive micropapillary carcinoma (*n*= 5), mucinous carcinoma (*n*=4), metaplastic carcinoma (*n*=4), invasive tubular carcinoma (*n*= 3), invasive apocrine carcinoma (*n*= 3), neuroendocrine carcinoma (*n*=1), invasive carcinoma with squamous differentiation (*n*=1).

^e^ Scarff-Bloom-Richardson grade system.

ER, estrogen receptor; PR, progesterone receptor.

Gross and histopathologic examinations of surgical specimens were performed by staff pathologists. Cross-sections of formalin-fixed paraffin-embedded specimens were obtained at 5-mm intervals perpendicular to the line connecting the nipple and the tumor center. Specimens were examined microscopically using hematoxylin and eosin staining. Lesion sizes were measured and largest tumor diameter was defined as tumor size.

Histopathologic diagnosis revealed invasive ductal carcinoma (IDC) in 86%, invasive lobular carcinoma (ILC) in 4.5%, invasive papillary carcinoma in 2.1%, and othersin 7.4%. The parameters used for multidetector computed tomography (MDCT) (Somatom Sensation 16 or 64 scanners, Siemens Medical Solutions, Forchheim, Germany) examinations were as follows: 100–120 kVp, 170 mAs, 5-mm collimation, a 10 mm/s table feed with a 1-s rotation time, 5.0-mm slice thickness, and 2.5–5.0 mm intervals. Synthetic sagittal and coronal images were reformatted at intervals of 3 mm and fully covered the region from anterior skin to the back of the chest. The effective dose for chest CT was 4.0 mSv, based on a standard patient model using an anthropomorphic phantom, and a conversion factor of 0.017 mSv/(mGy.cm) was used to convert dose-length product to effective dose [6]. Prior to chest CT, all patients fasted for >6 h. Iopromide (120 mL; Ultravist; Bayer Schering Pharma, Berlin) was injected intravenously at a rate of 4 mL/s, and CT images were obtained 30–40 s later. Our institutional review board approved this retrospective study, and the requirement for informed patient consent was waived.

### Image analysis

A subspecialty-trained chest radiologist and a third-year radiology resident retrospectively measured maximum diameters of breast tumors on CT images by consensus and compared these with pathologic results. CT determined tumor sizes were also analyzed for concordance with pathologic tumor sizes.

Concordance between CT and pathologic diameters was defined as a tumor size difference of <5 mm. When a CT measured size was >5 mm smaller than pathologic tumor size, CT determined tumor size was considered an underestimation, and conversely, when CT size was >5 mm larger than pathologic tumor size, it was considered an overestimation. In addition, concordance rates between IDC and non-IDC patients and between patients with a CT determined tumor diameter of <20 mm (Group 1) and ≥20 mm (Group 2) were compared.

### Statistical analysis

Maximum chest CT and pathology determined tumor sizes were compared by linear regression and Spearman’s rho correlation analysis. The chi-squared test was used to analyze concordances rates between chest CT and pathology with respect to tumor size and histologic type. Statistical significance was accepted at the 95% confidence level (*P*<0.05). A commercially available software program was used for data processing and analysis (PASW, version 17.0; SPSS, Chicago, IL, USA).

## Results

Overall, longest tumor diameters as determined by chest CT (average, 21 mm, range, 2–74 mm) and pathology (average, 22 mm; range, 3–90 mm) were not significantly different (*P* = 0.059)*,* and a linear relation was found between the two (pathology determined tumor size = 1.086 × CT determined tumor size - 1.141; Spearman’s rho correlation coefficient, 0.84; *P*<0.001).

Tumor sizes were concordant in 228 of the 285 patients (80%). However, 36 tumors (12.7%) were underestimated by CT by an average of 12 mm (range, 6–36 mm) and 21 tumors (7.4%) were overestimated by an average of 10 mm (range, 6–19 mm). The concordance rate was higher for tumors of <20 mm (88.5% *vs*. 71.9% for tumor size <20 mm *vs*. tumor size ≥20 mm, *P*<0.001) and for IDC (82.4% *vs*. 65.0% for IDC *vs*. non-IDC, *P =* 0.011) (Table 2).

**Table 2:** Comparison of CT and pathologically determined tumor sizes

		**Size (median ± SD)**		***Concordance with CT *(%)**	***Discordance with CT *(%)**	
	***n *(%)**	**CT**	**Pathology**		***Overestimated by CT***	***Underestimated by CT***
Overall	285 (100)	19±10	20±13	228 (80)	21 (7)	36 (13)
CT group^a^						
Group 1 (<20 mm)	139 (49)	15±5	13±4	123 (88)	15 (11)	1 (1)
Group 2 (**≥**20 mm)	146 (51)	27±10	30±13	105 (72)	6 (4)	35 (24)
Tumor type^b^						
IDC	245 (86)	20±10	20±12	202 (83)	18 (7)	25 (10)
Non-IDC	40 (14)	18±11	19±14	26 (65)	3 (8)	11 (28)

^a^ Comparison of group 1 to group 2, *P*<0.001.

^b^ Comparison across tumor types, *P* = 0.011.

## Discussion

A number of imaging modalities are used to assess tumor size and extent in breast cancer to determine optimal treatment. Mammography and ultrasonography are the first choices for the screening and diagnosis of breast cancer, but mammography is limited in premenopausal women with dense breasts and ultrasonography is not reliable enough to visualize tumor extensions when tumors exhibit extensive intraductal spread [7]. Breast magnetic resonance imaging (MRI) has attracted much attention recently because its ability to detect breast lesions sufficient to allow the accurate visualization of intraductal lesions around main tumors [7,8]. However, although MRI offers the advantage of sensitivity, it tends to overestimate tumor size and is limited by a relatively high false-positive rate, and thus, requires additional biopsies and increases patient anxiety, time, and costs [9-11]. In addition, MRI cannot be performed in patients with claustrophobia and is usually performed in the prone position, whereas surgery is performed with the patient supine. ChestCT examinations, on the other hand, are performed in the supine position, and thus, the positions of breasts in chest CT images better matches the surgical approach. Furthermore, due to the technological improvements offered by MDCT, several authors have concluded that CT of the breast provides an accurate preoperative means of assessing tumor extent and size [8,12-14]. However, breast CT in these reports had different acquisition technique with chest CT and is rarely used in present clinical practice, because the field of view is limited to the breast region and examinations require multiple scans, including precontrast CT scan and postcontrast CT scan at 70 to 100 s after contrast administration [13,15,16], or a dynamic acquisition technique (1, 3, and 8 min after contrast administration), which inevitably increases radiation dose [17]. In previous studies [17-19], the radiation dose required for breast CT was reported to be 28 mSv, which is approximately 10 times greater than that used for standard mammographic examinations (2.8 mSv), and seven times higher than that required for a chest CT examination.

For the assessment of tumor size in breast cancer, several authors have addressed the accuracies of mammography, ultrasonography (US), and magnetic resonance imaging (MRI) [20-24]; Ramirez *et al*. [22] concluded that generally, breast mammography determined tumor size correlated better with histopathologic size than MRI or US determined tumor sizes. However, MRI is regarded to be more accurate than mammography or ultrasonography in the ILC subtype, which tends to underestimate the sizes of ILC in up to 70%, and 80% of cases, respectively [23,24]. However, to the best of our knowledge, no previous study has addressed tumor size estimation by chest CT in breast cancer patients.

Because the incidence of metastasis is low and false-positive findings are more common than true-positive findings [4,5], the current National Comprehensive Cancer Network (NCCN) guidelines do not recommend the routine use of chest CT in operable breast cancer. Nevertheless, chest CT exanimations are being increasingly used in breast cancer to evaluate pulmonary and hepatic metastases [5,23,24]. Somewhat surprisingly, no report has been issued regarding the predictive value of chest CT for the evaluation of tumor size in breast cancer. In the present study, we found a positive correlation between chest CT and pathologically determined tumor sizes and that tumors sizes differed by <5 mm in 80% of our cohort. Furthermore, the concordance rate between CT and pathologic findings was higher for breast tumors of <20 mm and for those of the IDC histologic subtype.

Although current guidelines and available evidence do not support the routine preoperative chest CT evaluation of operable breast cancer, in some patients with a respiratory or abdominal symptom or who deny other imaging modalities, chest CT could provide an alternative means of evaluating tumor size, especially when the tumor is <20 mm of the IDC subtype.

This study had several limitations that warrant consideration. First, it is inherently limited by its retrospective design. In particular, the study is subject to selection bias, because women were selected for chest CT at the discretion of a breast surgeon, and breast cancers not visualized by chest CT were not included in the study. Second, we did not compare CT determined tumor sizes with ultrasonography, mammography, or breast MRI determined sizes, because not all patients underwent ultrasonography, mammography, or MRI, and because imagings were performed at different times. A prospective study is required to compare the abilities of chest CT, ultrasonography, mammography, and breast MRI to determine breast cancer size. Third, formalin fixation affects solid tissue measurements. Pritt and colleagues [25] observed 4% of breast cancer specimens decreased in size after overnight formalin fixation and that 40% shrank after processing and mounting.

## Conclusions

The present study shows that chest CT can be used to predict breast tumor size reliably in patients with breast cancer. In fact, chest CT determined breast tumor sizes were found to be well correlated with pathology-determined sizes, and for most, the size difference was <5 mm. In addition, the concordance rate between chest CT and pathology was found to be greater for breast tumors of <20 mm and for the IDC subtype.

## Abbreviations

IDC: Invasive ductal carcinoma; ILC: Invasive lobular carcinoma; MDCT: Multi-detector computed tomography; MRI: Magnetic resonance imaging; NCCN: National Comprehensive Cancer Network; US: Ultrasonography.

## Competing interests

The authors declare that they have no competing interests. The study had no external funding.

## Authors’ contributions

SJA, YSK, and EYK conceived the study and participated in the literature search, writing the manuscript, and editing. In addition, YSK and EYK participated in submission of the manuscript. HKP, EKC, YKK, YMS, and H-YC participated in study design, data analysis, manuscript writing, and editing. All the authors read and approved the final manuscript.
